# Enhanced Dissipation of Non-Steroidal Anti-Inflammatory Drugs (NSAIDs) in Soil by the Bioaugmentation with Newly Isolated Strain *Acinetobacter johnsonii* MC5

**DOI:** 10.3390/ijms26010190

**Published:** 2024-12-29

**Authors:** Mariusz Cycoń, Agnieszka Żmijowska, Magdalena Klim

**Affiliations:** 1 Department of Microbiology, Faculty of Pharmaceutical Sciences, Medical University of Silesia, Jagiellońska 4, 41-200 Sosnowiec, Poland; magdalena.klim@sum.edu.pl; 2 Laboratory of Analytical Chemistry, Ecotoxicology Research Group, Łukasiewicz Research Network—Institute of Industrial Organic Chemistry Branch Pszczyna, Doświadczalna 27, 43-200 Pszczyna, Poland; zmijowska.agnieszka@gmail.com

**Keywords:** *Acinetobacter johnsonii*, NSAIDs, ibuprofen, diclofenac, naproxen, bioaugmentation, soil bioremediation

## Abstract

The presented study investigated the possibility of using the *Acinetobacter johnsonii* MC5 strain, isolated from raw sewage by the enrichment culture method, in the bioremediation of soil contaminated with selected NSAIDs, i.e., ibuprofen (IBF), diclofenac (DCF), and naproxen (NPX), using the bioaugmentation technique. The degradation potential of *A. johnsonii* MC5 was first evaluated using a mineral salt medium containing drugs as the only sources of carbon and energy. The results show that the strain MC5 was capable of utilizing the tested compounds in medium, indicating that the drugs might be metabolically degraded. IBF and NPX were degraded with a similar rate and DT50 values were determined to be approximately 5 days, while the degradation process for DCF was slower, and the DT50 value was about 5 times higher (22.7 days) compared to those calculated for IBF and NPX. Bioaugmentation of non-sterile soil with *A. johnsonii* MC5 increased the rate of disappearance of the tested drugs, and DT50 values decreased 5.4-, 3.6-, or 6.5-fold for IBF, DCF, or NPX, respectively, in comparison with the values obtained for the soil with indigenous microorganisms only. The obtained results suggest that *A. johnsonii* MC5 may have potential for use in bioremediation of NSAID-contaminated soils; however, detailed studies are needed before using this strain in such process on a larger scale.

## 1. Introduction

The most popular NSAIDs include, but are not limited to, ibuprofen (IBF), diclofenac (DCF), and naproxen (NPX). Due to the availability of NSAIDs without a medical prescription, it is difficult to estimate the real levels of consumption of these drugs [1]. In addition, their use as auxiliary drugs during the COVID-19 pandemic contributed significantly to the global consumption of NSAIDs [2], and thus to the increase in their concentration in the environment [3]. NSAIDs in living organisms are not degraded, but only slightly transformed, so that they are excreted from the body in a practically unchanged form. When excreted from the body, they reach the sewage system and, at a later stage, the wastewater treatment plants (WWTPs). As shown by studies conducted, WWTPs are not able to remove the entire amount of NSAIDs from wastewater. NSAIDs and their metabolites accumulate in sewage sludge. Such sludge is used as fertilizer, and from there, NSAIDs can reach surface water, soil, and even drinking water, re-entering living organisms where they accumulate [4,5,6]. Another source of NSAIDs in the environment is incorrect storage and disposal of expired or unused drugs. These drugs in their pure form enter WWTPs or landfills, and from there they may reach groundwater and soil [7,8,9]. This may lead to an increase in their concentration in the environment being a serious danger for the maintenance of the ecological balance of various ecosystems [10,11]. To date, no parameters or limits have been set for NSAIDs in the soil environment. Available studies indicate that the concentration of NSAIDs varies in different countries and depends on the type of environmental sample. NSAIDs have been detected in soils in a wide range of concentrations, supporting the fact that many factors, such as frequency of sludge application, soil properties, precipitation, and runoff, may affect their presence in this environment [12]. As reported by Aznar et al. [13], the concentration of IBF and NPX in the soil was determined at the level of 15 and 59 µg/kg soil, respectively. In contrast, other NSAIDs, such as ketoprofen and diclofenac, were not found in the tested soil. In another study, the concentrations of NSAIDs in soil were determined at higher levels and were 610, 257, and 199 mg/kg soil for IBF, DCF, and NPX, respectively [14]. NSAIDs introduced into the soil may be adsorbed in soil particles, leached into groundwater or runoff into surface water [15,16], and undergo various processes involving physical, chemical, or biochemical transformations [17,18]. One of the most important mechanisms of the dissipation of NSAIDs in soil is the processes in which are involved microorganisms, and the natural microflora of soils has a specific potential to degrade drugs [19,20]. However, environmental conditions and factors such as moisture, temperature, and soil type strongly affect the degradation rate of these chemicals [15,21,22].

Extremely important issues are the processes of biodegradation and self-cleaning of the environment, in which microorganisms play a dominant role [15,23,24]. The attention of researchers is focused on the possibility of using strains of bacteria or fungi in the processes of bioremediation of the environment contaminated with various chemical compounds. As has been shown in many studies, microorganisms can degrade NSAIDs by either the metabolic pathway, using them directly as a source of carbon and energy, or the co-metabolic pathway, with an external source of the readily degradable organic compound. Metabolic or co-metabolic capabilities for degradation of NSAIDs have been confirmed for fungal strains from the genera *Phanerochaete* [25,26], *Trametes versicolor* [27,28], and *Cunninghamella* [29], and bacterial strains from the genera *Sphingomonas* [30], *Bacillus* [31], *Pseudomonas* [32,33], *Nocardia* [34], *Delftia* [33], *Rhodococcus* [35], *Patulibacter* [36], *Variovorax* [37], *Planococcus* [38], and *Stenotrophomonas* [39,40]. However, the above-mentioned cases were related to studies for the determination of the degradation potential of bacterial and/or fungal strains under laboratory conditions using various liquid media. In contrast, there are no reports on the use of microorganisms in the bioremediation of NSAID-contaminated soils. The soil ecosystem, due to the activity of microorganisms, is characterized by a specific potential to neutralize the effects of NSAID pollution and is able to degrade the introduced drugs; however, only to a limited level [13,15,19,21,23]. Currently, there are many methods used to clean up the soil environment, and among them, bioaugmentation, belonging to “green technologies”, is of great importance and is used to remove organic pollutants. Bioaugmentation is recommended mainly in places where the number of indigenous microorganisms to degrade contaminants is insufficient and/or where indigenous populations do not have the capacity required to metabolize contaminants [23]. In the case of soil, bioaugmentation involves increasing the catabolic potential of the soil microbial community to degrade contaminants. This can be achieved by the inoculation of soil with selected individual bacterial and/or fungal strains or consortia of them with preferred catabolic capabilities [23,24]. Therefore, the main purpose of the present study was to isolate a bacterial strain capable of degrading selected NSAIDs and determine its potential to remove drugs from contaminated soil using the bioaugmentation technique. The stated goal was realized by determining the degradation rates of ibuprofen, diclofenac, and naproxen in liquid medium and soil inoculated with the bacterial strain. The contribution of natural microflora to the degradation of tested NSAIDs in the soil and their dissipation under abiotic conditions were also determined.

## 2. Results

### 2.1. Identification of Bacterial Strain

**Figure 1:**
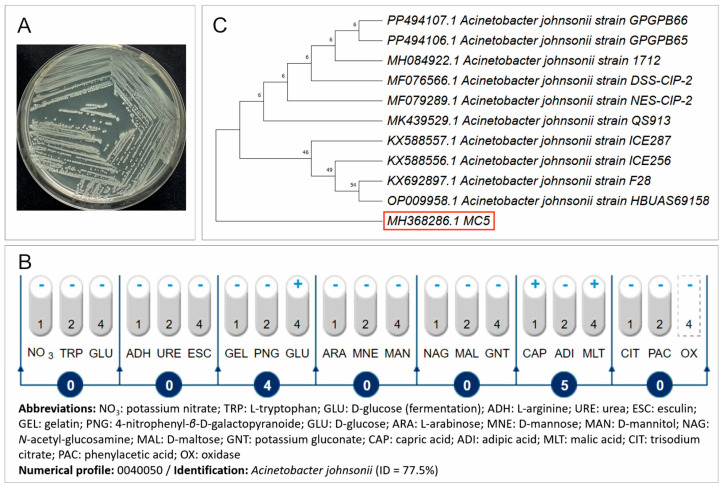
Culture on nutrient agar (**A**), the biochemical profile based on the API 20 NE system (**B**) and the phylogenetic tree based on the neighbor-joining method (**C**) of the isolated strain MC5. The cytochrome oxidase (OXI) probe, shown in the dotted box, was performed using the OXItest from Erba Lachema (Brno, Czech Republic). The phylogenetic position of the MC5 strain and its accession number in the GenBank are placed in a red rectangle. Bootstrap values from 1000 replications are indicated at the branches. For each strain is given the GenBank accession number.

The screening procedure allowed the isolation of a bacterial strain from the raw sewage in the presence of the applied NSAIDs, i.e., ibuprofen, diclofenac, and naproxen, which was designated as MC5. The isolated strain belongs to bacteria with low growth requirements and grows very well at 33 ± 2 °C on standard microbiological media, such as nutrient agar (Figure 1A), and possesses characteristic biochemical properties (Figure 1B). An analysis of the 16S rRNA gene sequence showed that strain MC5 is a member of the genus *Acinetobacter* with a similarity to the species *Acinetobacter johnsonii* (Figure 1C). The sequence of strain MC5 was submitted to the GenBank under accession number MH368286 (Figure S1).

### 2.2. Degradation of NSAIDs in Liquid Medium

#### 2.2.1. Degradation of Ibuprofen

**Figure 2:**
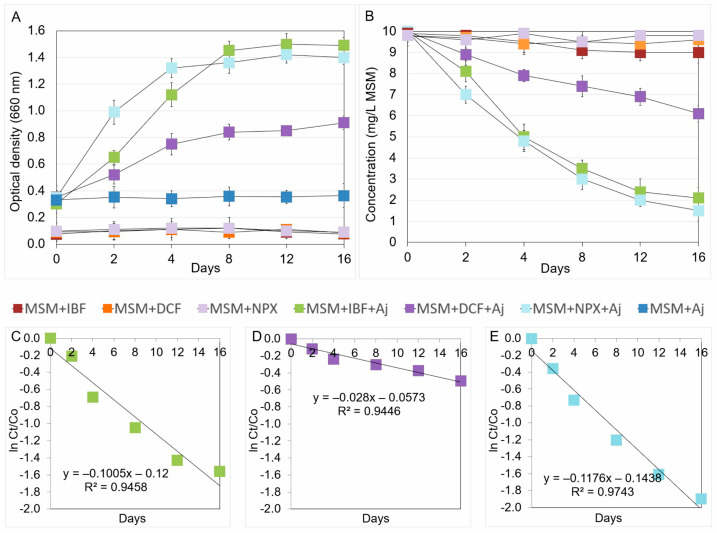
Data related to the growth rate of *Acinetobacter johnsonii* strain MC5 (**A**), concentrations of the analyzed NSAIDs (**B**), and the regression between ln(*C*_t_/*C*_0_) of the chemical data and time (**C**–**E**) obtained for the experiment with liquid medium. The data presented are the means of three replicates with standard deviations. Aj: *Acinetobacter johnsonii* MC5; *C*_0_: concentration of NSAID at time zero; *C*_t_: concentration of NSAID at time t; DCF: diclofenac; IBF: ibuprofen; ln: natural logarithm; MSM: mineral salt medium; and NPX: naproxen.

The growth dynamics of the *Acinetobacter johnsonii* MC5 strain in mineral salt medium determined by measuring absorbance (OD 660 nm) are shown in Figure 2A. The most intensive growth of the strain in the ibuprofen-supplemented medium (MSM+IBF+Aj) was recorded on the 12th day of incubation, after which the culture reached a stationary phase. In contrast, the control samples for the strain (MSM+Aj) and ibuprofen (MSM+IBF) showed no changes in optical density during the 16-day incubation period (Figure 2A). The growth of bacteria in MSM with ibuprofen reflected the rate of degradation of the test drug (Figure 2B). It was found that the rate of degradation of ibuprofen in the culture with *Acinetobacter johnsonii* MC5 (MSM+IBF+Aj) was 79% of the initial dose of the drug after 16 days of incubation. The data obtained from the regression plot (Figure 2C) and kinetics (Table 1) show that the degradation process of ibuprofen in MSM+IBF+Aj was characterized by a rate constant of *k* = 0.654/day, and the DT50 value calculated from the regression equation was determined to be 5.7 days. On the other hand, in the control without bacterial inoculum (MSM+IBF), ibuprofen was slightly degraded, and 91% of the initial dose of the introduced drug was determined at the end of the experiment (Figure 2B).

#### 2.2.2. Degradation of Diclofenac

The growth dynamics of the MC5 strain in MSM are presented in Figure 2A. The most intensive growth of the strain in medium supplemented with diclofenac (MSM+DCF+Aj) was determined after 16 days of incubation. In contrast, both controls, for the MC5 strain (MSM+Aj) and diclofenac (MSM+DCF) showed no changes in optical density, which was at a similar level during the incubation period. Data obtained from the experiment show that the degradation rate of diclofenac by *Acinetobacter johnsonii* MC5 (MSM+DCF+Aj) was 39% of the introduced drug dose after 16 days of incubation (Figure 2B). The regression plot (Figure 2D) and kinetics (Table 1) show that diclofenac degradation in MSM with strain MC5 was characterized by a rate constant of *k* = 0.300/day and a DT50 value of 22.7 days. The abiotic control (MSM+DCF) showed a low degradation rate of diclofenac at the level of 2% of the initial drug dose (Figure 2B).

#### 2.2.3. Degradation of Naproxen

The growth dynamics of the MC5 strain in MSM is presented in Figure 2A. The most intensive growth of the strain in medium supplemented with naproxen (MSM+NPX+Aj) was determined after 12 days of incubation. In contrast, both controls, for the MC5 strain (MSM+Aj) and naproxen (MSM+NPX) showed no changes in optical density, which was at a similar level during the incubation period. Data obtained from the experiment show that the degradation rate of naproxen by *Acinetobacter johnsonii* MC5 (MSM+NPX+Aj) was 85% of the introduced drug dose after 16 days of incubation (Figure 2B). The regression plot (Figure 2E) and kinetics (Table 1) show that naproxen degradation in MSM with strain MC5 was characterized by a rate constant of *k* = 0.685/day and a DT50 value of 4.7 days. On the other hand, the abiotic control (MSM+NPX) showed a low degradation rate of naproxen, at the level of 2% of the initial drug dose (Figure 2B).

### 2.3. Degradation of NSAIDs in Soil

#### 2.3.1. Degradation of Ibuprofen

**Figure 3:**
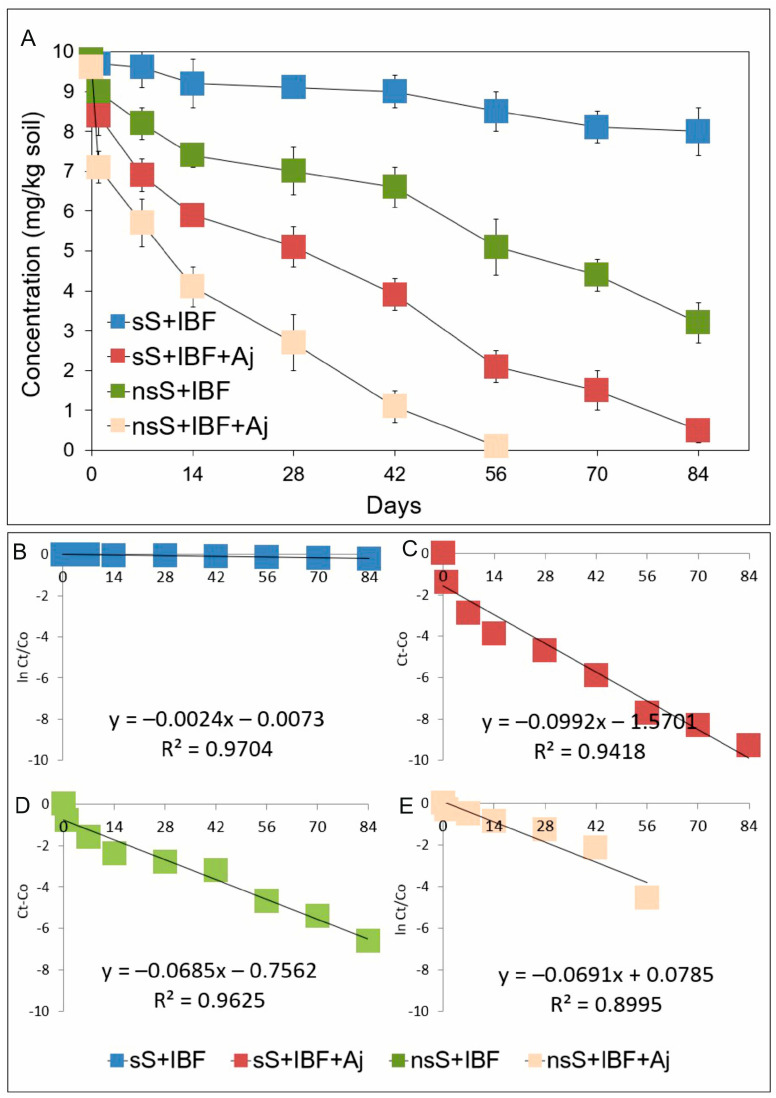
Data related to concentrations of ibuprofen (**A**) and the regression between ln(*C*_t_/*C*_0_) and *C*_t_ − *C*_0_ of the chemical data and time (**B**–**E**) obtained for the experiment with soil. The data presented are the means of three replicates with standard deviations. Aj: *Acinetobacter johnsonii* strain MC5; *C*_0_: concentration of NSAID at time zero; *C*_t_: concentration of NSAID at time t; IBF: ibuprofen; ln: natural logarithm; nsS: non-sterile soil; and sS: sterile soil.

The process of ibuprofen degradation in sterile soil inoculated with the *Acinetobacter johnsonii* MC5 strain (sS+IBF+Aj) was characterized by a rate constant of *k* = 0.266/day (Table 2). After 84 days of incubation, ibuprofen was found to be degraded at 94,9% of its initial concentration (Figure 3A), and the DT50 value, determining the time after which half the ibuprofen dose was degraded and calculated from the regression equation (Figure 3C), was determined to be 33.7 days (Table 2). In contrast, for the non-sterile sample with the bacterial strain (nsS+IBF+Aj), the drug degradation process was relatively faster, as evidenced by the higher value of the rate constant (*k* = 0.460/day) and the lower DT50 value (11.2 days) (Table 2). In contrast, the degradation of ibuprofen in non-sterile soil (nsS+IBF) was characterized by a rate constant of *k* = 0.166/day and a DT50 value of 60.5 days (Table 2). After 84 days of soil incubation, a drug degradation rate of 67.3% of the initial concentration was recorded (Figure 3A). The control sample for ibuprofen degradation under abiotic conditions showed a slow degradation process of the drug, which characterized a rate constant of *k* = 0.032/day (Table 2), and at the end of the incubation period, 81.6% of the initial dose of the drug was determined (Figure 3A).

#### 2.3.2. Degradation of Diclofenac

**Figure 4:**
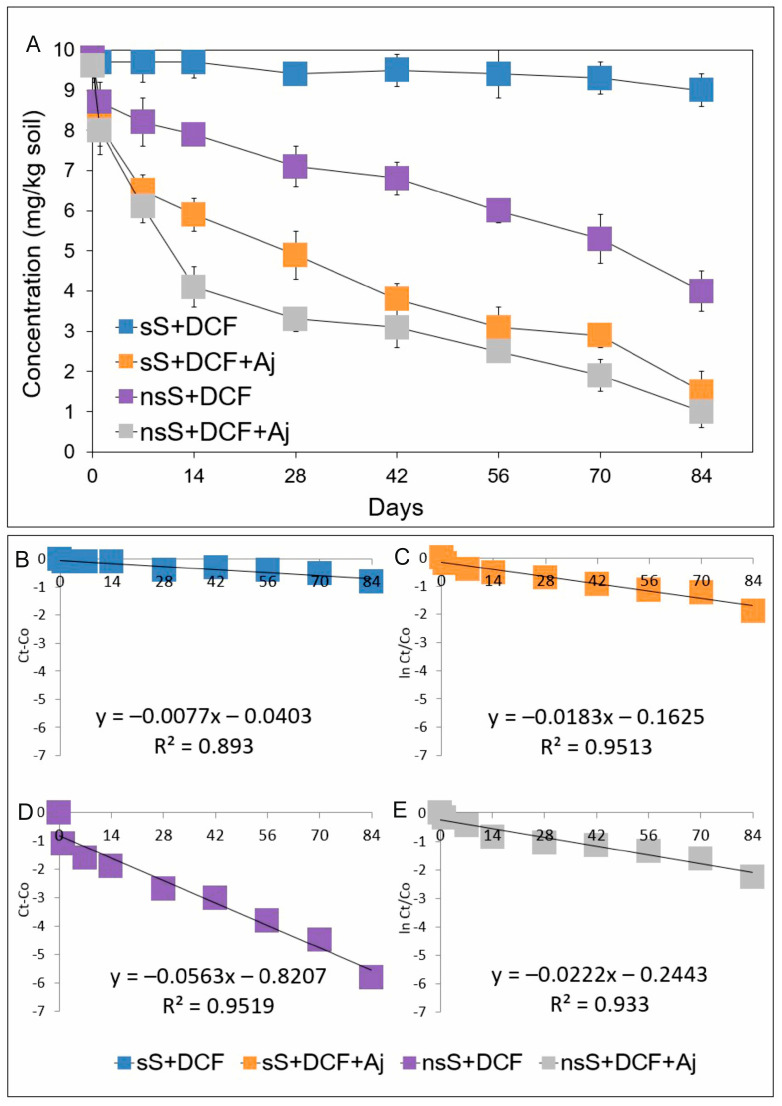
Data related to concentrations of diclofenac (**A**) and the regression between ln(*C*_t_/*C*_0_) and *C*_t_ − *C*_0_ of the chemical data and time (**B**–**E**) obtained for the experiment with soil. The data presented are the means of three replicates with standard deviations. Aj: *Acinetobacter johnsonii* strain MC5; *C*_0_: concentration of NSAID at time zero; *C*_t_: concentration of NSAID at time t; DCF: diclofenac; ln: natural logarithm; nsS: non-sterile soil; and sS: sterile soil.

The results show varying dynamics of diclofenac degradation in the soil, depending on both the conditions used and the bacterial strain (Figure 4A). The slowest degradation rate was recorded for sterile soil (sS+DCF). After 84 days of the test, diclofenac degradation was achieved at 8.2% of the initial concentration, and the degradation process was characterized by a rate constant of *k* = 0.019/day (Table 2). In the case of sterile soil inoculated with the *Acinetobacter johnsonii* MC5 strain (sS+DCF+Aj), the degradation rate was faster compared to sterile soil without inoculation, and at the end of the incubation period, the degradation rate of diclofenac was 84.7% of the initial concentration (Figure 4A), and the value for the disappearance time of 50% of the initial concentration (DT50) reached 29 days (Table 2). The results for the degradation of diclofenac in non-sterile soil (nsS) clearly show that the indigenous microflora was characterized by degradation capabilities. Introduced into the soil, diclofenac was degraded at a rate of 59.2% within 84 days of the experiment (Figure 4A), and the degradation process was characterized by a rate constant of *k* = 0.187/day. The DT50 value, calculated from the regression equation (Figure 4D), was 72.5 days (Table 2). Inoculation of non-sterile soil with the *A. johnsonii* MC5 strain resulted in an acceleration of the rate of diclofenac degradation compared to non-sterile soil without inoculum, and the introduced drug disappeared at 89.8% over the course of the study (Figure 4A). In this case, the degradation process was characterized by a rate constant of *k* = 0.297/day. The DT50 value, calculated from the regression equation (Figure 4E), was determined to be 20.2 days (Table 2).

#### 2.3.3. Degradation of Naproxen

**Figure 5:**
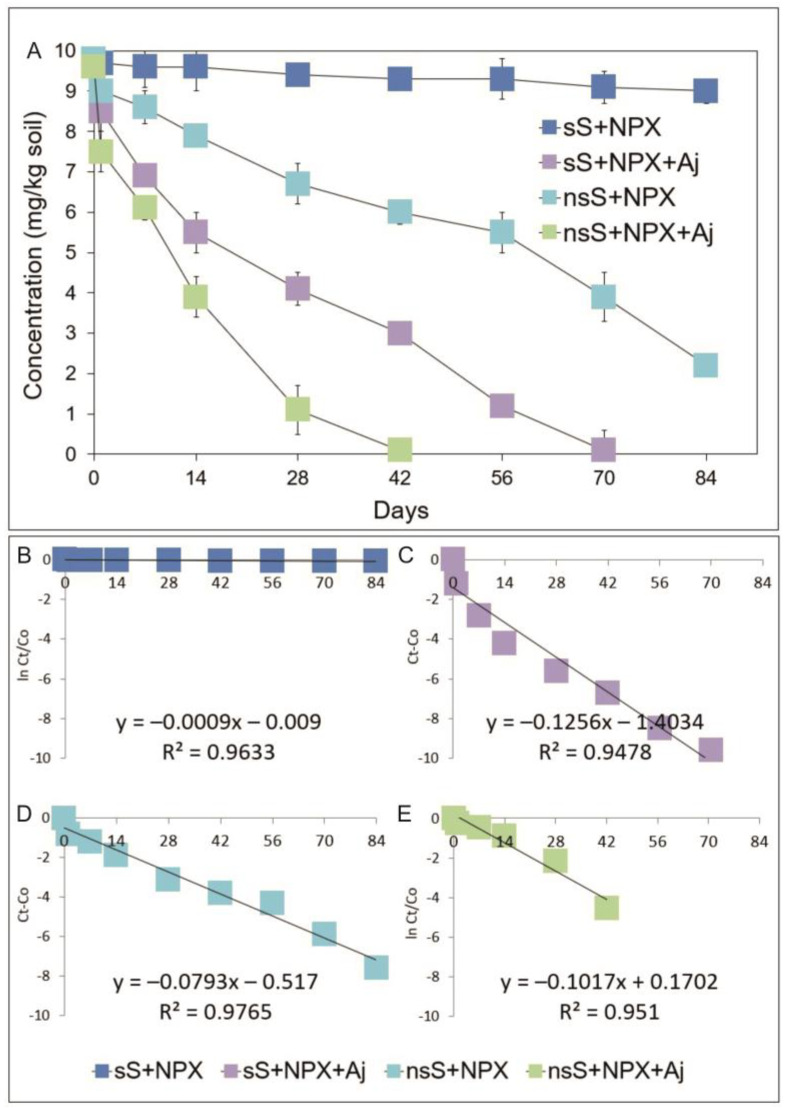
Data related to concentrations of naproxen (**A**) and the regression between ln(*C*_t_/*C*_0_) and *C*_t_ − *C*_0_ of the chemical data and time (**B**–**E**) obtained for the experiment with soil. The data presented are the means of three replicates with standard deviations. Aj: *Acinetobacter johnsonii* strain MC5; *C*_0_: concentration of NSAID at time zero; *C*_t_: concentration of NSAID at time t; ln: natural logarithm; NPX: naproxen; nsS: non-sterile soil; and sS: sterile soil.

As for the other drugs tested, naproxen also showed varying dynamics of disappearance depending on the conditions used (Figure 5A). The slowest degradation rate was recorded for sterile soil (sS). After 84 days of the test, naproxen degradation was achieved at 8% of the initial concentration, and the degradation process was characterized by a rate constant of *k* = 0.020/day (Table 2). In the case of sterile soil inoculated with the *Acinetobacter johnsonii* strain (sS+NPX+Aj), the degradation rate was faster compared to sterile soil without inoculation, and at the end of the incubation period, the degradation rate of naproxen was almost 100% of the initial concentration (Figure 5A), and the value for the disappearance time of 50% of the initial concentration (DT50) reached 27.4 days (Table 2). The results for the degradation of naproxen in non-sterile soil (nsS+NPX) clearly show that the indigenous microflora was characterized by degradation capabilities. Introduced into the soil, naproxen was degraded at a rate of 78% within 84 days of the experiment (Figure 5A), and the degradation process was characterized by a rate constant of *k* = 0.171/day. The DT50 value, calculated from the regression equation (Figure 5), was 55.3 days (Table 2). Inoculation of non-sterile soil with *Acinetobacter johnsonii* strain MC5 (nsS+NPX+Aj) resulted in an acceleration of the rate of naproxen degradation compared to non-sterile soil without inoculum (nsS+NPX), and the introduced drug disappeared at 100% over the course of the study. In this case, the degradation process was characterized by a rate constant of *k* = 0.577/day. The DT50 value, calculated from the regression equation (Figure 5E), was determined to be 8.5 days (Table 2).

## 3. Discussion

The screening procedure using enriched cultivation allowed the isolation of bacterial strain MC5 identified as *Acinetobacter johnsonii* and characterized by a specific degradation potential in relation to selected NSAIDs, i.e., ibuprofen, diclofenac, and naproxen. In general, bacteria from genus *Acinetobacter*, known as very metabolically active bacteria, characterize a broad spectrum of biochemical activities related to the ability to degrade various natural and synthetic compounds and were isolated from various environments contaminated with different chemicals [41,42,43,44,45,46,47,48,49]. In the present study, the strain MC5 was capable of utilizing the tested compounds in mineral salt medium as carbon and energy sources for growth, indicating that the drugs might be metabolically degraded. However, the rate of degradation of individual compounds varied. Ibuprofen and naproxen were degraded with a similar rate and the DT50 values were determined to be <5 days, while the degradation process for diclofenac was slower, and the DT50 value was about five times higher (22.7 days) compared to those calculated for ibuprofen and naproxen. The observed differences in the level of degradation of NSAIDs might be due to the different chemical structure and physicochemical properties of the individual drugs.

As reported in the literature, there is no information on bacteria belonging to *Acinetobacter johnsonii* capable of degrading all the NSAIDs used in this study, i.e., ibuprofen, diclofenac, and naproxen. However, previous studies have shown the ability of strains belonging to the genus *Acinetobacter* to degrade other drugs, i.e., antibiotics. For example, a study by Wang et al. [50] demonstrated the ability of the *Acinetobacter* sp. strain to degrade sulfamethoxazole, sulfadiazine, and sulfamethazine, while such properties of the tested strain were not observed in relation to other antibiotics, i.e., trimethoprim, triclosan, and carbamazepine, and in addition to NSAID, i.e., diclofenac. Another study showed that the *Acinetobacter calcoaceticus* strain T32 was capable of degrading the antibiotic furazolidone in the medium (5 mg/L) within a 5-day incubation period [51]. However, other studies have shown varied rates of NSAIDs degradation by bacteria belonging to different genera. For example, the *Bacillus thuringiensis* B1 strain was characterized as being able to degrade 5 mg/L ibuprofen in 2 days and a low amount of naproxen [31]. However, the authors found that the drugs used did not provide sufficient sources of carbon and energy for the growth of the tested bacterial strain. In contrast, Murdoch and Hay [37] described bacterial strains *Sphingomonas* Ibu-2 and *Variovorax* Ibu-1, which were capable of degrading ibuprofen at high concentrations. In another study, the *Stenotrophomonas maltophila* strain KB2 was capable of degrading 78% of the applied dose of naproxen in the presence of glucose [40]. In turn, exposure of the *Klebsiella* sp. strain KSC to diclofenac at a high concentration (70 mg/L) resulted in mineralization of the drug within 72 h [52]. Other studies demonstrated the ability of bacteria from the genus *Pseudomonas* to degrade various drugs. For example, Żur et al. [53] evaluated the degradation potential and effect of DCF on the *Pseudomonas moorei* strain KB4. As shown in the study, the strain KB4 degraded DCF in monosubstrate culture at a concentration of 0.5 mg/L, but the addition of glucose and sodium acetate into the medium increased degraded doses of DCF to 1 mg/L in 12 days.

In our study, we did not determine the formed metabolites, and the degradation potential of the MC5 strain in the mineral medium was assessed by the decrease in concentrations of parent compounds during incubation. However, other studies reported the formation of various metabolites during the transformation of NSAIDs by bacterial strains. As revealed by the research of Wojcieszyńska et al. [40], *Stenotrophomonas maltophilia* KB2, in medium with the addition of glucose or phenol, transformed naproxen by its hydroxylation to 5,7,8-trihydroxynaproxen that can be cleaved by hydroxyquinol 1,2-dioxygenase, and the cleavage product is probably further oxidatively cleaved by gentisate 1,2-dioxygenase. In turn, Murdoch and Hay [30] identified isobutylcatechol, 5-formyl-2-hydroxy-7-methylocta-2,4-dienoic acid, and 2-hydroxy-5-isobutylhexa-2,4-dienoic acid as metabolites of the ibuprofen transformation by *Sphingomonas* Ibu-2 in the monosubstrate medium. In a study of Marchlewicz et al. [31], 2-hydroxyibuprofen was detected in a culture of *Bacillus thuringiensis* B1 with ibuprofen and an additional carbon source in the form of glucose. In the same study, the authors also identified *O*-desmethylnaproxen during the transformation of naproxen by strain B1. In turn, hydroxydiclofenac, dihydroxydiclofenac, trihydroxydiclofenac, tetrahydroxydiclofenac, and decarboxylated derivatives were identified as metabolites in a study on the transformation of diclofenac by *Klebsiella* sp. KSC [52].

The results show varying dynamics of the disappearance of the tested NSAIDs in the soil, depending on both the conditions used and the bacterial strain. In general, pharmaceuticals in soil undergo various physical, chemical, and biochemical processes [18,22,54]. However, the fate and persistence of individual NSAIDs in soil will depend on a number of factors, such as the physicochemical properties of the compound and the characteristics of the environment [15]. In general, the present study showed that naproxen had the highest susceptibility to degradation and diclofenac the lowest, regardless of the conditions used. The slowest degradation rate for all NSAIDs tested was recorded for sterile soil. It was also shown that the natural soil microflora had the ability to degrade the introduced drugs at a concentration of 10 mg/kg of soil, with DT50 values ranging from 55.3 days for naproxen to 72.5 days for diclofenac. Other authors also confirmed that microorganisms are primarily involved in the degradation of NSAIDs in soil. For example, Al-Rajab et al. [55], found that diclofenac, when added to soils of different textures, was rapidly mineralized without delay, with a half-life of <5 days. In another study, the degradation of selected NSAIDs in soil, i.e., naproxen, diclofenac, and ibuprofen, was found to be relatively fast with a maximum half-life of 20.44 days, with the latter drug showing the slowest degradation [19]. Additionally, a study by Shu et al. [56] demonstrated low to moderate persistence in soil for IBF and NPX, with half-lives ranging from 4.9 to 14.8 days, while KTF was found to be highly persistent with an average half-life of 33 days. As shown in earlier studies, the rate of degradation of NSAIDs in non-sterile soil is also strongly dependent on their concentration. It was reported that diclofenac, ibuprofen, and naproxen at a concentration of 0.05 mg/kg soil were rapidly degraded in 0.2 to 9.5 days, while a significant extension of the degradation rate, up to 68 days, was observed when the drugs were used at a concentration of 5 mg/kg soil [57]. Similarly, Xu et al. [19] showed that the degradation rate constant for naproxen, diclofenac, and ibuprofen decreased as their initial soil concentrations increased, suggesting that microbial activity was inhibited at high levels of the drugs. However, after applying a relatively high dose of drugs in our study, no lag phase was observed, suggesting that the drugs used did not have an effect on decreasing the activity of the indigenous microorganisms.

The study also demonstrated the ability of the *Acinetobacter johnsonii* MC5 strain to degrade all tested NSAIDs in sterile soil; however, their rates of degradation varied and the DT50 values obtained were 33.7, 29.0, and 27.4 days for ibuprofen, diclofenac, and naproxen, respectively. In general, the strain MC5 introduced into sterile soils showed a higher degradation potential for NSAID removal than those observed for non-sterile soil with only indigenous microorganisms, and in addition, no lag phases were observed. These results can be explained by the fact that the inoculated strain had earlier contact with NSAIDs and could, therefore, adapt faster to the drugs than natural microflora of the soil with no contact with NSAIDs. This phenomenon was also observed in studies on degradation of other contaminants [58,59]. Bioaugmentation of non-sterile soil with a strain of *Acinetobacter johnsonii* increased the rate of disappearance of the tested drugs, and DT50 values decreased 5.4-, 3.6-, or 6.5-fold for ibuprofen, diclofenac, or naproxen, respectively, in comparison with the values obtained for the soil with natural microflora only. This confirms that the introduced strain increased the catabolic potential of the natural microflora. In our study, we used an inoculum of 1.6 × 10^7^ cells/g of soil, and as the results show, this inoculum appears to be sufficient for the degradation of the NSAIDs tested. Although the survival ability of the introduced MC5 strain was not directly monitored in the present study, it can be concluded indirectly from the results that the introduced strain had survival capabilities. For all drugs tested, in both sterile and non-sterile soil, the MC5 strain showed higher degradation potential compared to non-sterile soil with only natural microflora. Previous studies demonstrated that the size of the inoculum is an important factor for the effective biodegradation of different chemicals that contaminate soil, such as antibiotics, pesticides, or petroleum compounds [59,60,61,62]. It has been observed that an inoculum with a density below 10^4^ cells/g soil was not effective in the degradation of pesticides due to the low survival rate of the introduced bacteria. For example, Singh et al. [63] demonstrated that *Enterobacter* sp. did not degrade the pesticide chlorpyrifos when introduced into soil below an inoculum density of 10^3^ cells/g soil. Therefore, the use of an inoculum of sufficient density can compensate for the initial decrease in the number of introduced bacteria, and the remaining ones that survive will be able to degrade contaminants [64,65].

The results obtained from the degradation of tested NSAIDs in non-sterile soil might not only be related to the characteristics of the microbial community of the soil used, but also to a high degree to its physicochemical properties. The soil used in the study was classified as sandy loam with a high sandy fraction and low organic matter content, which might result in a lower affinity of NSAIDs for soil particles and hence a higher availability of the drugs for the microorganisms responsible for their degradation [66,67,68]. A study by Chefetz et al. [54] showed that both the content and physicochemical nature of soil organic matter strongly influenced the sorption of diclofenac and naproxen. Additionally, Shu et al. [56] demonstrated that the degradation of naproxen, ketoprofen, and ibuprofen in the soil with alkaline-treated biosolids was inhibited in comparison with the soil without supplementation. In contrast, the addition of biosolids to sterile or non-sterile soil did not accelerate the dissipation of diclofenac. A similar effect was also observed in relation to other pharmaceuticals [15,69], as well as to other organic contaminants such as polycyclic aromatic hydrocarbons [70,71] and pesticides [72,73]. In addition, the degradation rate of the tested NSAIDs might also be related to their chemical structure and physicochemical properties determining toxicity to soil microorganisms [74,75] and susceptibility to degradation processes [19,38,76]. The availability of NSAIDs for microorganisms may be limited due to their low solubility, thus also reducing their susceptibility to biodegradation. This low solubility also contributes to the long-term persistence of these drugs in soil [19,69]. In general, the slowest degradation rate in our study was recorded for diclofenac, which is characterized by the lowest solubility in water at 2.4 mg/L among the NSAIDs tested. In contrast, the highest degradation rate was found for naproxen, whose solubility in water (16 mg/L) is almost 7-fold greater in comparison with the solubility of diclofenac. In general, diclofenac has higher affinity for soil organic matter, which results in a lower susceptibility to degradation and a longer persistence in the soil environment [54].

The results obtained in the present study are difficult to compare with the findings of other authors due to the lack of previous studies on the use of bacteria in the bioremediation of NSAID-contaminated soils. On the other hand, previous studies confirmed the usefulness of bioaugmentation in the process of the remediation of soils contaminated with antibiotics, pesticides, or aromatic hydrocarbons [77,78]. However, the use of bacteria that degrade NSAIDs and other organic compounds to remediate contaminated soil may raise some difficulties, as little is known about the fate of inoculants in the soil. The ability to survive and compete with indigenous microorganisms, as well as the degradative activity of inoculants, are key factors that may limit the effectiveness of the bioremediation process [77,78]. However, detailed studies on the interactions between this strain and the soil environment and its ability to survive and compete with the native microflora are needed before using this strain in bioremediation on a larger scale.

## 4. Materials and Methods

### 4.1. Chemicals

**Figure 6:**
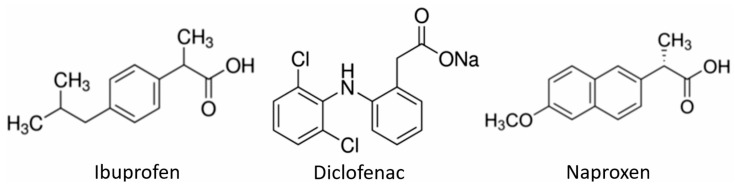
Chemical structure of NSAIDs used in the experiment.

Three different European Pharmacopoeia (EP) reference standards of NSAIDs obtained from Merck (Darmstadt, Germany) and commercially available NSAIDs were used in the experiment. In the first case, they were ibuprofen (IBF, α-methyl-4-(isobutyl)phenylacetic acid), diclofenac sodium (DCF, 2-[(2,6-dichlorophenyl)amino]benzeneacetic acid sodium salt), and naproxen (NPX, (*S*)-(+)-2-(6-methoxy-2-naphthyl)propionic acid). In the second case, they were Ibuprom (ibuprofen 200 mg and additional ingredients)(US Pharmacia, Wrocław, Poland), Diclac^®^ Duo 150 (diclofenac sodium salt 150 mg and additional ingredients)(INPHARM/SANDOZ, Warszawa, Poland), and Naproxen 500 (naproxen 500 mg and additional ingredients)(Polfarmex, Kutno, Poland). Chemical structures of NSADs used in the experiment are shown in Figure 6. In addition, the composition of commercially available NSAIDs provided by manufacturers is presented in Table S1. All other chemicals and solvents were high-performance liquid chromatography (HPLC) grade and obtained from Merck.

### 4.2. Isolation, Cultivation, and Identification of Bacterial Strain

The source of the bacterial strain capable of degrading selected drugs was raw sewage collected from the municipal sewage treatment plant “Gigablok” located in Katowice-Szopienice, southern Poland. Isolation of the bacterial strain involved a two-step enrichment procedure, during which mineral salt medium (MSM) was used. The composition of MSM is presented in Appendix A.

**Figure 7:**
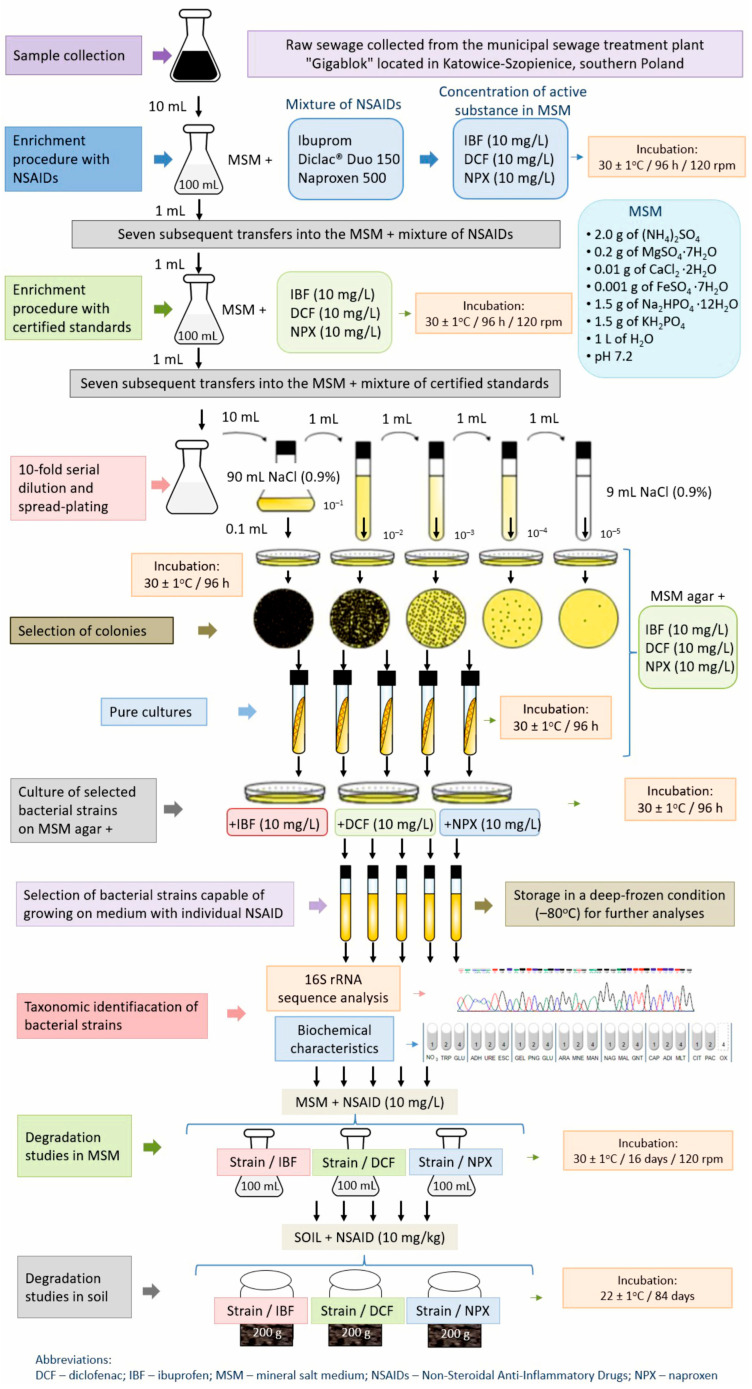
Test procedures used in the experiment including isolation and identification of bacterial strain and degradation studies of selected NSAIDs in liquid medium and soil.

The first stage of isolation involved enriched cultivation in MSM supplemented with a mixture of commercially available NSAIDs (Figure 7). For this purpose, 10 mL of raw sewage was added to 300 mL flasks with 100 mL of MSM supplemented with a mixture of Ibuprom, Diclac^®^ Duo 150, and Naproxen 500. Drugs were added in such a quantity that the final concentration of each active substance, i.e., IBF, DCF, and NPX in the medium was 10 mg/L. The use of commercial drug preparations in the first stage of the screening procedure of the bacterial strain was intended to reflect the natural conditions under which discarded expired drugs enter the environment, including raw sewage, and to cause adaptation of the bacterial strain to use the pure active substances of individual preparations as carbon and energy sources in the next stage of the procedure. Samples were incubated for 96 h on a rotary shaker (120 rpm) in a darkened thermostatic chamber maintained at 30 ± 1 °C. After this time, 1 mL of suspension was transferred into flasks containing the fresh medium supplemented with the same concentration of commercially available NSAIDs and incubated for an additional 96 h under the same conditions.

After seven subsequent transfers into the same medium, a second isolation phase involved enriched cultivation in MSM with a mixture of reference standards of the tested drugs (Figure 7). For this purpose, 1 mL of suspension from the previous stage was added to 300 mL flasks with 100 mL of MSM supplemented with mixture of IBF, DCF, and NPX, whose concentration in the medium was 10 mg/L each. Samples were incubated for 96 h on a rotary shaker (120 rpm) in a darkened thermostatic chamber maintained at 30 ± 1 °C. After this time, 1 mL of suspension was transferred into flasks containing the fresh medium supplemented with the same concentration of reference standards of NSAIDs and incubated for an additional 96 h under the same conditions.

After seven subsequent transfers into the same medium, serial dilutions of the flask samples were plated onto MSM agar plates supplemented with a mixture of IBF, DCF, and NPX (each 10 mg/L) for isolation of individual colonies (Figure 7). Plates were incubated for 96 h in a darkened thermostatic chamber maintained at 30 ± 1 °C. Isolates exhibiting distinct colonial morphologies were isolated by repeated streaking on the same agar medium. Finally, a strain capable of growing on a mineral medium containing each of the individual drugs tested was selected. The use of high concentrations of drugs (10 mg/L) during the screening procedure was aimed to isolate a bacterial strain with a really high potential to degrade NSAIDs, and whose growth would not be limited by high doses of the drugs tested.

The next step in the procedure involved characterization and identification of the isolate using a biochemical test and 16S rRNA gene analysis (Figure 7). The biochemical properties of the isolate and the substrate utilization pattern were determined using an API 20NE System (bioMérieux Inc., Marcy l’Etoile, France) according to the manufacturer’s recommendations. For the 16S rRNA sequence analysis, the genomic DNA was extracted from a strain collected at the late exponential stage of growth using a GeneMATRIX Bacterial and Yeast Genomic DNA Purification Kit (Eurx, Gdańsk, Poland) as described in the protocol of the manufacturer. The 16S rRNA gene of the isolate was amplified using the PCR mix containing the universal primer pair: 27f and 1492r [79] obtained from Sigma-Aldrich (Steinheim, Germany) [80] and other components (Table S2). Amplification was performed using a PCR Master Mix Kit (Promega, Madison, WI, USA) according to the manufacturer’s recommendations and a PTC-118 Thermal Cycler (Bio-Rad, Hercules, CA, USA) under the defined conditions (Table S3).

After amplification, the products were purified with a GeneMATRIX PCR/DNA Clean-Up Purification Kit (Eurx) according to the protocol of the manufacturer before the amplicons were sequenced. Gene sequencing was performed using a Big Dye^®^ Terminator Cycle Sequencing Kit (Applied Biosystem, Waltham, MA, USA) and an AbiPrism^®^3100 Genetic Analyzer. The obtained sequence was compared to known 16S rRNA gene sequences using the BLAST server at the National Center for Biotechnology Information (NCBI; http://www.ncbi.nlm.nih.gov/) (accessed on 20 May 2018). DNA sequences were aligned using CLUSTAL W. Phylogenetic analysis was performed by the neighbor-joining (NJ) method using the MEGA ver. 11 software.

A characterized and identified bacterial strain capable of growing on MSM agar in the presence of each of the tested drugs was used for further studies that included the degradation of drugs in liquid medium and soil. The test procedures used in the experiment, including isolation and identification of bacterial strain and degradation studies of selected NSAIDs in liquid medium and soil, are shown in Figure 7.

### 4.3. Study of the Degradation of NSAIDs

#### 4.3.1. Study of the Degradation of NSAIDs in Liquid Medium

**Figure 8:**
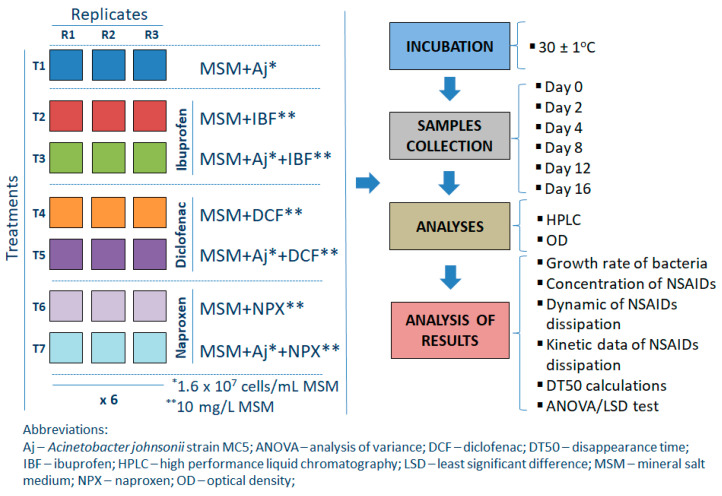
Design and performed analyses for the experiment with mineral salt medium.

Degradation studies were performed in 200 mL Erlenmeyer flasks containing 100 mL of sterile MSM containing the individual reference standards of NSAIDs as the sole carbon source and inoculated with bacterial strain (MSM+Aj+IBF, MSM+Aj+DCF, and MSM+Aj+NPX). Samples of MSM+Aj and MSM+IBF or DCF or NPX were used as controls. Drugs were added in such a quantity that their final concentrations in the medium were 10 mg/L. The bacterial suspension was introduced into the suitable MSM samples in order to produce a final bacterial count of approximately 1.6 × 10^7^ cells/L MSM. A detailed description of the inoculum preparation for the experiment with MSM is provided in Appendix B. The experiment had a completely randomized block design and there were three replications of each treatment for each sampling time, which produced a total of 126 flasks in the experiment (i.e., seven treatments × three replications × six sampling times). Design and performed analyses for the experiment with MSM are shown in Figure 8, while a detailed description of the treatments used is provided in Appendix C.

Flasks were incubated on a rotary shaker (120 rpm) in a darkened thermostatic chamber maintained at 30 ± 1 °C. Samples were periodically removed (randomly) for the bacterial growth rate determination, as well as for chemical analyses to determine IBF, DCF, or NPX concentrations. The growth of bacteria was recorded spectrophotometrically by measuring the OD at 660 nm using a UV-VIS spectrophotometer (Varian, Palo Alto, CA, USA). Design and performed analyses for the experiment with MSM are shown in Figure 8.

#### 4.3.2. Study of the Degradation of NSAIDs in Soil

Soil samples were collected from the top layer (0–20 cm) from grass-covered fields located in the vicinity of Żywiec, southern Poland. In the laboratory, the soil was sieved to a maximum particle size of <2 mm and immediately used for the experiment. The properties of the soil were determined according to ISO standards as presented in Table 3, and it was classified as loamy sand according to US/FAO System [81].

**Figure 9:**
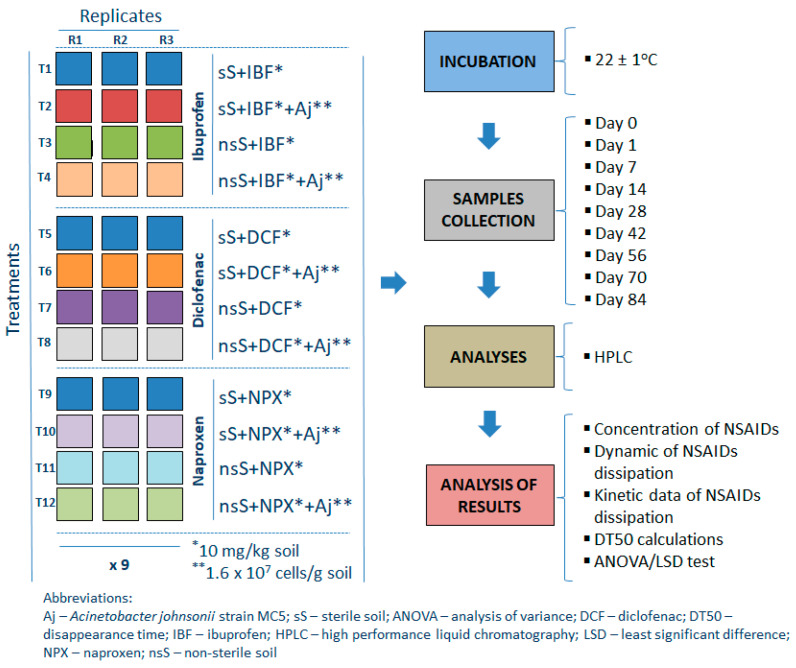
Design and performed analyses for the experiment with soil.

To determine the effect of abiotic and biotic conditions on the rate of disappearance of the drugs tested, sterile and non-sterile soil were used in the experiment, respectively. In the first case, soil was sterilized three times by autoclaving for 1 h at 121 °C one week prior to the commencement of the experiment to permit the release of the toxic volatile compounds produced. The experiment had a completely randomized block design and there were three replications of each treatment for each sampling time, which produced a total of 108 pots in the experiment (i.e., four treatments × three replications × nine sampling times) for each NSAID tested. Design and performed analyses for the experiment with MSM are shown in Figure 9, while a detailed description of the treatments used is provided in Appendix D.

In order to ensure an even distribution of NSAIDs in the soil, the individual reference standards were added to sterile quartz sand (<0.5 mm). Next, the mixture of sand (50 g/kg soil) and NSAID was added into the soil portion and thoroughly mixed. Drugs were added in such a quantity that their final concentrations in the soil were 10 mg/kg. The use of a high initial concentration of NSAIDs tested reflected the most adverse scenarios associated with the entry of large quantities of drugs into the soil as a result of the uncontrolled disposal of unused drugs into municipal waste or depositing them in landfills and was also intended to evaluate the potential hazards of these drugs on the degradation potential of naturally occurring soil microflora. The bacterial suspension was introduced into the suitable soil portion in order to produce a final bacterial count of approximately 1.6 × 10^7^ cells/g soil. A detailed description of the inoculum preparation for the experiment with MSM is provided in Appendix B.

The water content of the soils was adjusted to 50% of the maximum water holding capacity and checked every week. Throughout the incubation period, water losses exceeding 5% of the initial values were compensated for by the addition of sterile deionized water. The pots with soil samples were covered with perforated polypropylene sheets and were incubated in the dark at 22 ± 1 °C for 84 days. Samples (three replicates for each treatment) were periodically removed (randomly) from the test system for chemical analyses to determine NSAID concentrations (on days 0, 1, 7, 14, 28, 42, 56, 72, and 84). Design and performed analyses for the experiment with soil are shown in Figure 9.

### 4.4. Chemical Analyses

To determine the concentration of the tested NSAIDs in the MSM medium samples, the first step of the procedure was to add 0.1% solution (*v*/*v*) of formic acid (0.1 mL) to the sample (10 mL) to acidify it. Then, deionized water was added to obtain a final volume of 20 mL and extracted twice with ethyl acetate (10 mL) on a rotary shaker for 30 min. The obtained extracts were filtered through anhydrous Na_2_SO_4_, evaporated to dryness at 45 °C using a rotary evaporator (IKA, RV05 Basic, Janke & Kunkel-Ika Labortechnik, Staufen, Germany), and then the obtained dry residue was dissolved in 20 mL of a mixture of acetonitrile: 0.05% orthophosphoric acid solution (50:50, *v*/*v*) and subjected to chromatographic analysis.

To determine the concentration of the tested NSAIDs in the soil samples, the first step of the procedure was to add 0.1% solution (*v*/*v*) of formic acid (5 mL) to the soil sample (10 g) to acidify it. Then, ethyl acetate (15 mL) was added to the samples and ultrasonicated for 10 min, preceded by 5 min of shaking. In the next step, the obtained suspension was centrifuged, the solution from above the sediment was filtered through anhydrous Na_2_SO_4_, and the residue was re-extracted with sodium acetate (15 mL), shaken, centrifuged, and filtered. The combined extracts were evaporated to dryness at 45 °C using a rotary evaporator, and the obtained dry residue was dissolved in 20 mL of a mixture of acetonitrile: 0.05% orthophosphoric acid solution (50:50, *v*/*v*) and subjected to chromatographic analysis. The chromatographic system and conditions used for analysis are shown in Table 4.

The analytical method used in the determination of the tested NSAIDs was checked in its validation procedure for linearity, specificity, precision, recovery, limits of quantification (LOQ), and detection (LOD), and the data obtained are shown in Table 5. Calibration curves, chromatograms for NSAID standards, control and NSAID-treated MSM samples, and control and NSAID-treated soil samples obtained during the validation studies are presented in Figures S2–S4, Figure S5, Figure S6, and Figure S7, respectively.

### 4.5. Data Analysis

Based on the analysis of the kinetics of NSAID dissipation in MSM and soil, their degradation rates were fitted to the first-order or zero-order kinetic models. The rate constant *k* (day) was determined using the equation *C*_t_/*C*_0_ = e^−*kt*^ or *k* = 1/t (*C*_0_ − *C*_t_) for the first-order or zero-order kinetic models, respectively, where *C*_0_ is the amount of a drug in MSM or soil at time zero, and *C*_t_ is the amount of a drug in MSM or soil at time t (day). Times in which the NSAID concentrations in MSM or soils were reduced by 50% (DT50 values) were calculated from the linear equation obtained from the regression between ln(*C*_t_/*C*_0_) and *C*_t_ − *C*_0_ of the chemical data and time. The results from three replicates of each treatment were also evaluated using statistical analysis. The significance (*p* < 0.05) of differences for DT50 was assessed by post hoc comparison of means using the least significant differences (LSD) test using the Statistica 13.3 PL software package.

## 5. Conclusions

The screening procedure using the enrichment culture technique allowed for the isolation of bacterial strain MC5 identified as *A. johnsonii* and characterized by a specific degradation potential in relation to selected NSAIDs, i.e., ibuprofen, diclofenac, and naproxen. The strain MC5 was capable of utilizing the tested compounds in mineral salt medium as carbon and energy sources for growth, indicating that the drugs might be metabolically degraded. IBF and NPX were degraded with a similar rate and the DT50 values were determined to be approximately 5 days, while the degradation process for DCF was slower, and the DT50 value was about five times higher (22.7 days) compared to those calculated for IBF and NPX. The results show varying dynamics of the disappearance of the tested NSAIDs in the soil, depending on both the conditions used and the bacterial strain. In general, it was shown that NPX had the highest susceptibility to degradation and diclofenac the lowest, regardless of the conditions used. The observed differences in the level of degradation of NSAIDs might be due to the different chemical structure and physicochemical properties of the individual drugs. It was confirmed that the degradation of the tested drugs in the soil environment occurs under both abiotic and biotic conditions, with the dominant role played by microbial activity. Bioaugmentation of non-sterile soil with *A. johnsonii* MC5 increased the rate of disappearance of the tested drugs, and DT50 values decreased 5.4-, 3.6-, or 6.5-fold for IBF, DCF, or NPX, respectively, in comparison with the values obtained for the soil with indigenous microorganisms only. This confirmed that the introduced strain increased the catabolic potential of the natural microflora. The degradation and bioremediation abilities of the *A. johnsonii* MC5 strain in relation to all the NSAIDs used can be used in the remediation processes of contaminated soils.

## Figures and Tables

**Table 1:** Degradation rate constant (*k*) and DT50 values for the analyzed NSAIDs obtained from the experiment with mineral salt medium.

Treatment	*k* (Day)	DT50 *
MSM+IBF+Aj	0.654	5.7 ^b^
MSM+DCF+Aj	0.300	22.7 ^a^
MSM+NPX+Aj	0.685	4.7 ^b^

* DT50 values were calculated from the linear equation obtained from the regression between ln(*C*_t_/*C*_0_) of the chemical data and time shown in Figure 2C–E. Aj: *Acinetobacter johnsonii* strain MC5; DCF: diclofenac; IBF: ibuprofen; MSM: mineral salt medium; and NPX: naproxen. The data presented are the means of three replicates. Significant differences (LSD test, *p* < 0.05) between the values for treatments are marked with different letters.

**Table 2:** Degradation rate constant (*k*) and DT50 values for the analyzed NSAIDs obtained from the experiment with soil.

System	Treatment	*k* (Day)	DT50 *
IBF/Aj	sS+IBF	0.032	285.7 ^Ca^
	sS+IBF+Aj	0.266	33.7 ^Ac^
	nsS+IBF	0.166	60.5 ^Bb^
	nsS+IBF+Aj	0.460	11.2 ^Bd^
DCF/Aj	sS+DCF	0.019	631.1 ^Ba^
	sS+DCF+Aj	0.291	29.0 ^Bc^
	nsS+DCF	0.187	72.5 ^Ab^
	nsS+DCF+Aj	0.297	20.2 ^Ad^
NPX/Aj	sS+NPX	0.020	760.0 ^Aa^
	sS+NPX+Aj	0.288	27.4 ^Bc^
	nsS+NPX	0.171	55.3 ^Cb^
	nsS+NPX+Aj	0.577	8.5 ^Cd^

* DT50 vales were calculated from the linear equation obtained from the regression between ln(*C*_t_/*C*_0_) and *C*_t_ − *C*_0_ of the chemical data and time shown in Figure 3B–E, Figure 4B–E and Figure 5B–E. Aj: *Acinetobacter johnsonii* strain MC5; DCF: diclofenac; IBF: ibuprofen; NPX: naproxen; nsS: non-sterile soil; and sS: sterile soil. The data presented are the means of three replicates. Significant differences (LSD test, *p* < 0.05) between treatments within one system and between treatments from various systems are denoted with different lower and uppercase letters, respectively.

**Table 3:** Characteristics of the soil used in the experiment.

Parameter	Value *	Method of Determination
Sand (2000–50 μm) (%)	70.0 ± 2.2	ISO 11277:2020 [82]
Silt (<50–2 μm) (%)	22.0 ± 1.3	
Clay (<2 μm) (%)	8.0 ± 1.1	
Density g/cm^3^	1.23 ± 0.07	
pH (in water) (1:5)	6.8 ± 0.2	ISO 10390:2021 [83]
Cation exchange capacity (CEC) (cmol+/kg)	11.0 ± 0.7	ISO 11260:2018 [84]
Water holding capacity (WHC) (%)	38.0 ± 1.3	ISO 14239:2017 [85]
C_org_ (%)	1.2 ± 0.1	ISO 23400:2021 [86]
N_tot_ (%)	0.13 ± 0.02	ISO 11261:1995 [87]
Microbial biomass (mg/kg)	854 ± 16	ISO 14240-1:1997 [88]

* The values are the means (n = 3) ± SD, which was within 5% of the mean.

**Table 4:** Characterization of the chromatographic system and conditions used during the determination of the tested NSAIDs.

Parameter	Conditions/Value
Chromatographic system	HPLC
Instrument	Shimadzu, Prominence-i LC-2030C 3D (Shimadzu, Inc., Japan)
Column	Luna 5 µm C18 100A, l = 250 mm, ϕ = 4.6 mm
Mobile phase	acetonitrile: 0.05% solution of ortho-phosphoric acid (70:30, *v*/*v*)
Temperature	40 °C
Flow rate	1 mL/min
Volume of injection	20 µL
Wavelength	220 nm
Detection system	DAD
Retention time	NPX—5 min, DCF—10 min, IBF—16 min
Data analysis	LabSolution LC-2030C 3D software ver. 5.90

DAD: diode array detector; DCF: diclofenac; HPLC: high-performance liquid chromatography; IBF: ibuprofen; and NPX: naproksen.

**Table 5:** Data obtained from the validation of the analytical method used to determine the concentrations of the tested NSAIDs in MSM medium and soil.

Parameter		Determined NSAIDs		
		IBF	DCF	NPX
Range of linearity		0.025–10.0 µg/mL, i.e., 0.05–20.0 mg/L (kg)		
*R*^2^ for calibration curve		0.9994	0.9996	0.9999
LOQ		0.5 mg/L (kg)		
LOD		0.05 mg/L (kg)		
Recovery [%]/precision (RSD [%]) for MSM	LOQ	98.4/0.1	97.2/0.6	96.7/0.5
	10 × LOQ	99.2/0.2	98.1/0.3	97.9/0.1
Recovery [%]/precision (RSD [%]) for soil	LOQ	83.6/1.1	88.1/1.4	83.4/0.7
	10 × LOQ	97.5/0.1	93.0/0.2	86.9/0.1

DCF: diclofenac; IBF: ibuprofen; LOD: limit of detection; LOQ: limit of quantification; MSM: mineral salt medium; NPX: naproxen; RSD: relative standard deviation.

## Data Availability

The authors declare that the data supporting the findings of this study are available within the article and Supplementary Materials. All information are available from the corresponding author on reasonable request.

## Supplementary Materials

The following supporting information can be downloaded at: https://www.mdpi.com/article/10.3390/ijms26010190/s1.

## Appendix A

The medium contained 2.0 g of (NH_4_)_2_SO_4_, 0.2 g of MgSO_4_·7H_2_O, 0.01 g of CaCl_2_·2H_2_O, 0.001 g of FeSO_4_·7H_2_O, 1.5 g of Na_2_HPO_4_·12H_2_O, and 1.5 g of KH_2_PO_4_ per liter of deionized water. The final pH value was adjusted to 7.2. After autoclaving (121 °C, 20 min) and cooling, and the medium was supplemented with selected NSAIDs.

## Appendix B

In order to prepare the inoculum, the bacterial strain was cultured in 200 mL Erlenmeyer flasks containing 100 mL of nutrient broth (BTL, Poland). At the exponential phase, the bacteria were pelleted by centrifugation (5 min, 10,000× *g*). The pellet was washed twice with 0.85% of sterile NaCl and then resuspended in 0.85% of sterile NaCl to obtain a bacterial suspension at a concentration of approximately 2.1 × 10^9^ cells/mL. The cell density (OD 660 nm) was measured using a densitometer (Densimat^®^, bioMérieux, France). Next, the bacterial suspension was introduced into MSM or soil in order to produce a final bacterial count of approximately 1.6 × 10^7^ cells/mL MSM or g soil.

## Appendix C

The experiment with MSM had the following treatments:

- MSM+Aj—mineral salt medium with *Acinetobacter johnsonii* strain MC5 (1.6 × 10^7^ cells/mL): 18 flask × 100 mL; for analysis—3 replicates × 6 sampling times
- MSM+IBF—mineral salt medium with ibuprofen (10 mg/L): 18 flask × 100 mL); for analysis—3 replicates × 6 sampling times
- MSM+Aj+IBF—mineral salt medium with *Acinetobacter johnsonii* strain MC5 (1.6 × 10^7^ cells/mL) and ibuprofen (10 mg/L): 18 flask × 100 mL; for analysis—3 replicates × 6 sampling times
- MSM+DCF—mineral salt medium with diclofenac (10 mg/L): 18 flask × 100 mL; for analysis—3 replicates × 6 sampling times
- MSM+Aj+DCF—mineral salt medium with *Acinetobacter johnsonii* strain MC5 (1.6 × 10^7^ cells/mL) and diclofenac (10 mg/L): 18 flask × 100 mL; for analysis—3 replicates × 6 sampling times
- MSM+NPX—mineral salt medium with naproxen (10 mg/L): 18 flask × 100 mL; for analysis—3 replicates × 6 sampling times
- MSM+Aj+NPX—mineral salt medium with *Acinetobacter johnsonii* strain MC5 (1.6 × 10^7^ cells/mL) and naproxen (10 mg/L): 18 flask × 100 mL; for analysis—3 replicates × 6 sampling times

## Appendix D

The experiment with soil had the following treatments:

- sS+IBF—sterile soil with ibuprofen (10 mg/kg): 5400 g (27 pots × 200 g); for analysis—3 replicates × 9 sampling times
- sS+IBF+Aj—sterile soil with ibuprofen (10 mg/kg) and *Acinetobacter johnsonii* strain MC5 (1.6 × 10^7^ cells/g): 5400 g (27 pots × 200 g); for analysis—3 replicates × 9 sampling times
- nsS+IBF—non-sterile soil with ibuprofen (10 mg/kg): 5400 g (27 pots × 200 g); for analysis—3 replicates × 9 sampling times
- nsS+IBF+Aj—non-sterile soil with ibuprofen (10 mg/kg) and *Acinetobacter johnsonii* strain MC5 (1.6 × 10^7^ cells/g): 5400 g (27 pots × 200 g); for analysis—3 replicates × 9 sampling times
- sS+DCF—sterile soil with diclofenac (10 mg/kg): 5400 g (27 pots × 200 g); for analysis—3 replicates × 9 sampling times
- sS+DCF+Aj—sterile soil with diclofenac (10 mg/kg) and *Acinetobacter johnsonii* strain MC5 (1.6 × 10^7^ cells/g): 5400 g (27 pots × 200 g); for analysis—3 replicates × 9 sampling times
- nsS+DCF—non-sterile soil with diclofenac (10 mg/kg): 5400 g (27 pots × 200 g); for analysis—3 replicates × 9 sampling times
- nsS+DCF+Aj—non-sterile soil with diclofenac (10 mg/kg) and *Acinetobacter johnsonii* strain MC5 (1.6 × 10^7^ cells/g): 5400 g (27 pots × 200 g); for analysis—3 replicates × 9 sampling times
- sS+NPX—sterile soil with naproxen (10 mg/kg): 5400 g (27 pots × 200 g); for analysis—3 replicates × 9 sampling times
- sS+NPX+Aj—sterile soil with naproxen (10 mg/kg) and *Acinetobacter johnsonii* strain MC5 (1.6 × 10^7^ cells/g): 5400 g (27 pots × 200 g); for analysis—3 replicates × 9 sampling times
- nsS+NPX—non-sterile soil with naproxen (10 mg/kg): 5400 g (27 pots × 200 g); for analysis—3 replicates × 9 sampling times
- nsS+NPX+Aj—non-sterile soil with naproxen (10 mg/kg) and *Acinetobacter johnsonii* strain MC5 (1.6 × 10^7^ cells/g): 5400 g (27 pots × 200 g); for analysis—3 replicates × 9 sampling times
