# Supplementary material for: Enhanced Dissipation of Non-Steroidal Anti-Inflammatory Drugs (NSAIDs) in Soil by the Bioaugmentation with Newly Isolated Strain Acinetobacter johnsonii MC5
Source: Int J Mol Sci. 2024 Dec 29;26(1):190. doi: 10.3390/ijms26010190 (PMC11720006; doi:10.3390/ijms26010190)
Supplement: Supplementary file 1 [file ijms-26-00190-s001.zip › ijms-3345271-supplementary.pdf]

Supplementary Material Table 1. Composition of commercially available NSAIDs used in the experiment.

Pharmaceutical preparation	Contents
**Ibuprom**	ibuprofen 200 mg, cellulose, corn starch, guar gum, talc, crospovidone Type A, silica, colloidal hydrate, *hydrogenated vegetable oil, hydroxypropyl cellulose,* macrogol 400, gelatin, sucrose, caolin, confectioners sugar, calcium carbonate, acacia gum, titanium dioxide E171, Opalux White AS 7000, Carnauba wax, Opacode Black S-1-17823
**Diclac® Duo 150**	diclofenac sodium salt 150 mg, lactose monohydrate, calcium hydrogen phosphate dehydrate, microcrystalline cellulose, magnesium stearate, carboxymethyl starch sodium salt Type A, silica colloidal anhydrous, corn starch, iron oxide E172, hypromellose
**Naproxen 500**	naproxen 500 mg, methyl cellulose, croscarmellose sodium, magnesium stearate, silica colloidal anhydrous

Supplementary Material Table S2. Composition of PCR mix for amplification of the 16S rRNA encoding gene fragment.

Component	Volume [µL]	Concentration
5 × GoTaq Flexi Buffer (Promega, USA)	10	1 ×
$MgCl_2$, 25 mM (Promega, USA)	3	1.5 mM/µL
dNTP Mix, 25 mM (Blirt, Poland)	0.4	0.2 mM/µL
Primer 27f 10 µM (Sigma-Aldrich, Germany) (5'-AGA GTT TGA TCC TGG CTC AG-3')	2.5	0.5 µM/µL
Primer 1492r 10 µM (Sigma-Aldrich, Germany) (5'-TAC GGT TAC CTT GTT ACG ACT T-3')	2.5	0.5 µM/µL
GoTaq DNA Polymerase (5 U/µL) (Promega)	0,3	1.5 U/µL
DNA from bacteria	3	0.2 µg/µL
Nuclease-free water (Promega, USA)	28.3	-
Final volume	50	-

Supplementary Material Table S3. Reaction conditions for amplification of the 16S rRNA encoding gene fragment.

Phase	Temperature [°C]	Time [min]	Number of cycles
Initial denaturation	95	5	1
Denaturation	95	1	30
Attachment	54	30	30
Elongation	72	2	30
Final elongation	72	5	1
Cooling	4	90	1

```
LOCUS       MH368286                1281 bp    DNA     linear   BCT 25-MAY-2018
DEFINITION  Acinetobacter johnsonii strain MC5 16S ribosomal RNA gene, partial
            sequence.
ACCESSION   MH368286
VERSION     MH368286.1
KEYWORDS    .
SOURCE      Acinetobacter johnsonii
  ORGANISM  Acinetobacter johnsonii
            Bacteria; Pseudomonadota; Gammaproteobacteria; Moraxellales;
            Moraxellaceae; Acinetobacter.
REFERENCE   1  (bases 1 to 1281)
  AUTHORS   Cycon,M.
  TITLE     Direct Submission
  JOURNAL   Submitted (20-MAY-2018) Microbiology and Virology, Medical
            University of Silesia, Jagiellonska, Sosnowiec 41-200, Poland
COMMENT     ##Assembly-Data-START##
            Sequencing Technology :: Sanger dideoxy sequencing
            ##Assembly-Data-END##
FEATURES             Location/Qualifiers
     source          1..1281
                     /organism="Acinetobacter johnsonii"
                     /mol_type="genomic DNA"
                     /strain="MC5"
                     /isolation_source="raw sewage"
                     /db_xref="taxon:40214"
     rRNA            <1..>1281
                     /product="16S ribosomal RNA"
ORIGIN
        1 tagcggcgga cgggtgagta atgcttagga atctgcctat tagtgggggа caacattccg
       61 aaaggaatgc taataccgca tacgccctac gggggaaagc aggggatctt cggaccttgc
      121 gctaatagat gagcctaagt cagattagct agttggtggg gtaaaggcct accaaggcga
      181 cgatctgtag cgggtctgag aggatgatcc gccacactgg gactgagaca cggcccagac
      241 tcctacggga ggcagcagtg gggaatattg gacaatgggc gcaagcctga tccagccatg
      301 ccgcgtgtgt gaagaaggcc ttttggttgt aaagcacttt aagcgaggag gaggctactt
      361 ggattaatac tctaggatag tggacgttac tcgcagaata agcaccggct aactctgtgc
      421 cagcagccgc ggtaatacag agggtgcgag cgttaatcgg atttactggg cgtaaagcgt
      481 gcgtaggcgg ctttttaagt cggatgtgaa atccctgagc ttaacttagg aattgcattc
      541 gatactggga agctagagta tgggagagga tggtagaatt ccaggtgtag cggtgaaatg
      601 cgtagagatc tggaggaata ccgatggcga aggcagccat ctggcctaat actgacgctg
      661 aggtacgaaa gcatggggag caaacaggat tagataccct ggtagtccat gccgtaaacg
      721 atgtctacta gccgttgggg cctttgaggc tttagtggcg cagctaacgc gataagtaga
      781 ccgcctgggg agtacggtcg caagactaaa actcaaatga attgacgggg gcccgcacaa
      841 gcggtggagc atgtggttta attcgatgca acgcgaagaa ccttacctgg tcttgacata
      901 gtaagaactt tccagagatg gattggtgcc ttcgggaact tacatacagg tgctgcatgg
      961 ctgtcgtcag ctcgtgtcgt gagatgttgg gttaagtccc gcaacgagcg caaccctttt
     1021 ccttatttgc cagcgggtta agccgggaac tttaaggata ctgccagtga caaactggaa
     1081 ggaaggcggg gaacaacgtc aagtcatcat gggcccttac gaacaagggc taccaaccgt
     1141 gctacaatgg gccggtacaa aagggttgct accttagcga taggatgcta atctcaaaaa
     1201 agccgatcgt aattccggat tggaattctg caactcgact cccttgaagt ccggaatccc
     1261 ttagtaatcc cgggatccaa a
```

Supplementary Material Figure S1: The identification data of the MC5 strain from GenBank.

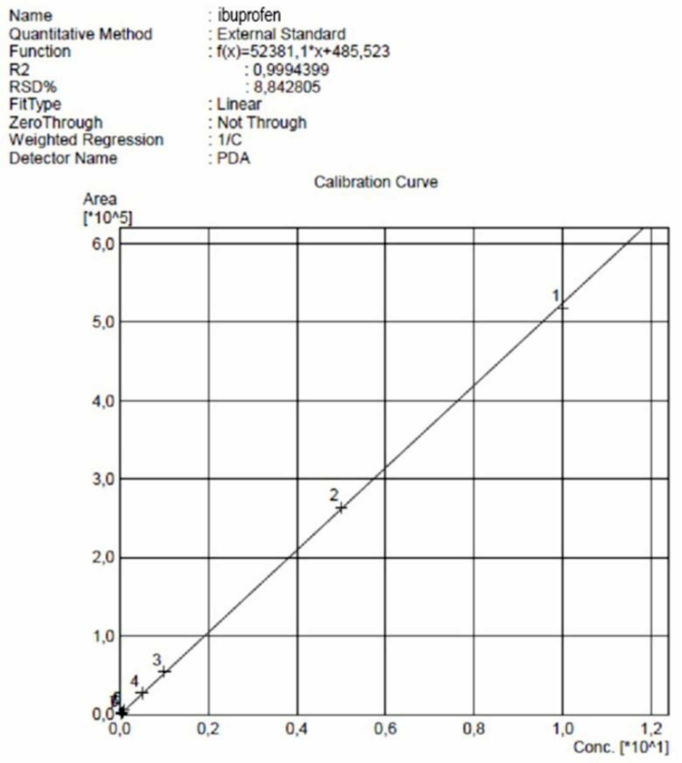

Supplementary Material Figure S2: Calibration curve for working solutions of ibuprofen standard in the concentration range of 0.025–10 μg/mL, obtained during the validation procedure of the analytical method used to determine NSAID concentrations in MSM and soil.

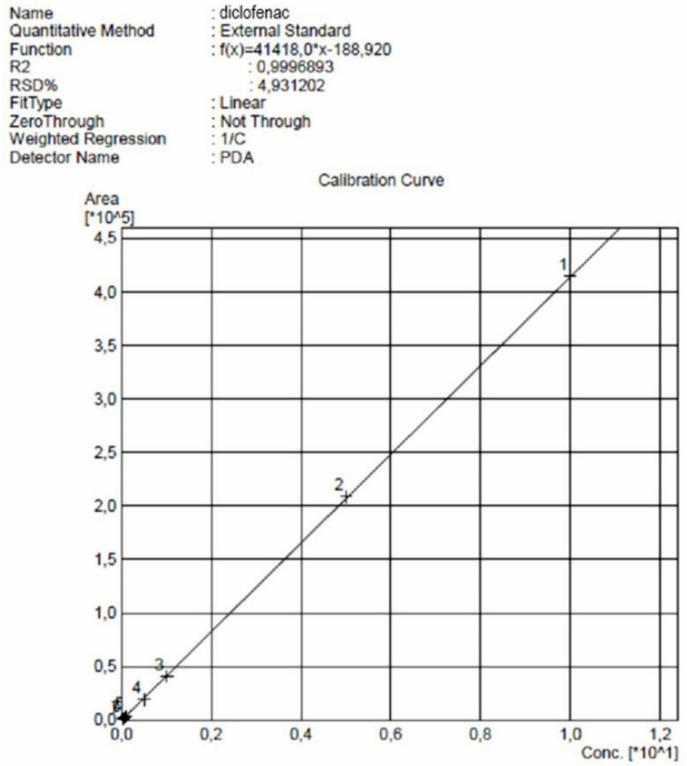

Supplementary Material Figure S3: Calibration curve for working solutions of diclofenac standard in the concentration range of 0.025–10 μg/mL, obtained during the validation procedure of the analytical method used to determine NSAID concentrations in MSM and soil.

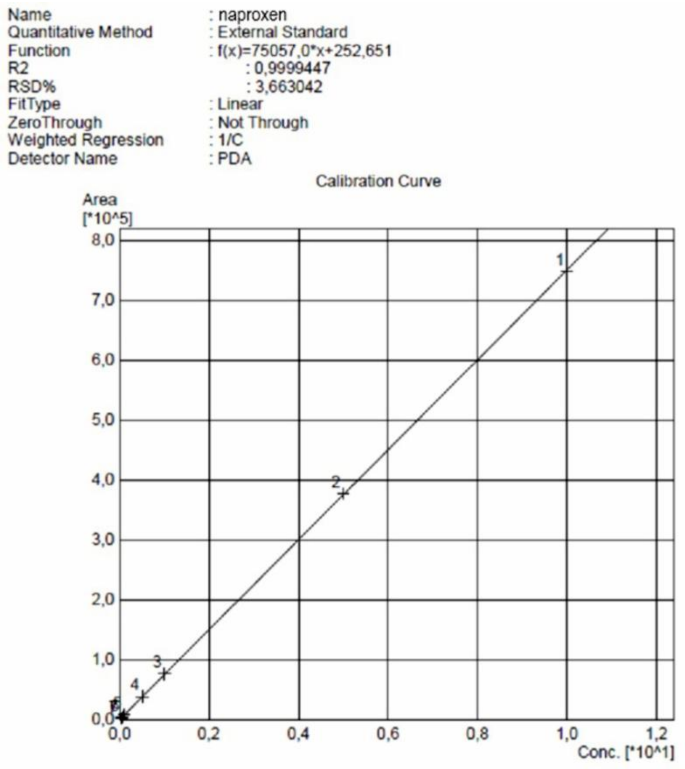

Supplementary Material Figure S4: Calibration curve for working solutions of naproxen standard in the concentration range of 0.025–10 μg/mL, obtained during the validation procedure of the analytical method used to determine NSAID concentrations in MSM and soil.

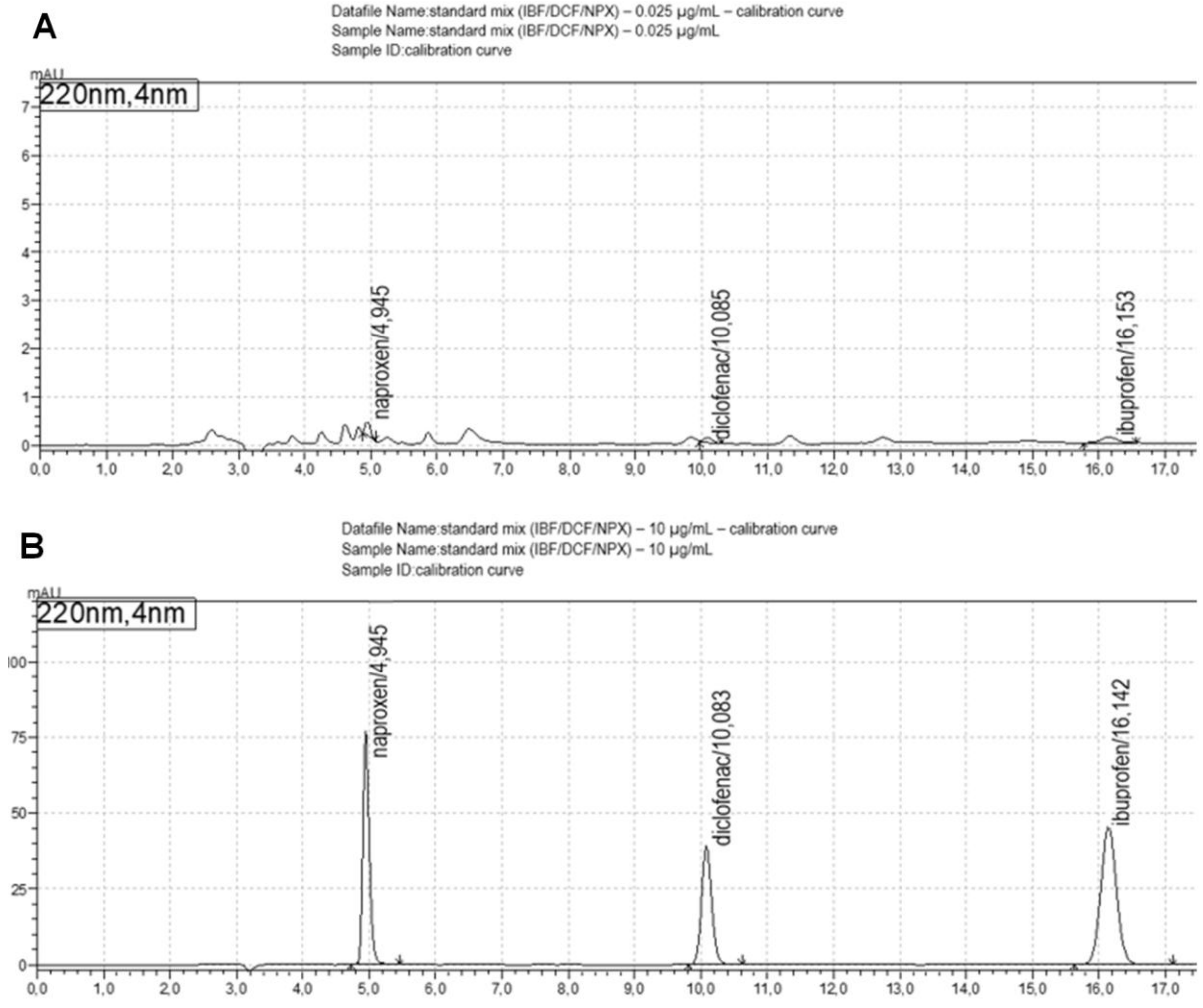

Supplementary Material Figure S5: Example chromatograms for working solutions of ibuprofen, diclofenac and naproxen standards at concentrations of 0.025 µg/mL (A) and 10 µg/mL (B) used to prepare the calibration curve, and obtained during the validation procedure of the analytical method used to determine NSAID concentrations in MSM and soil.

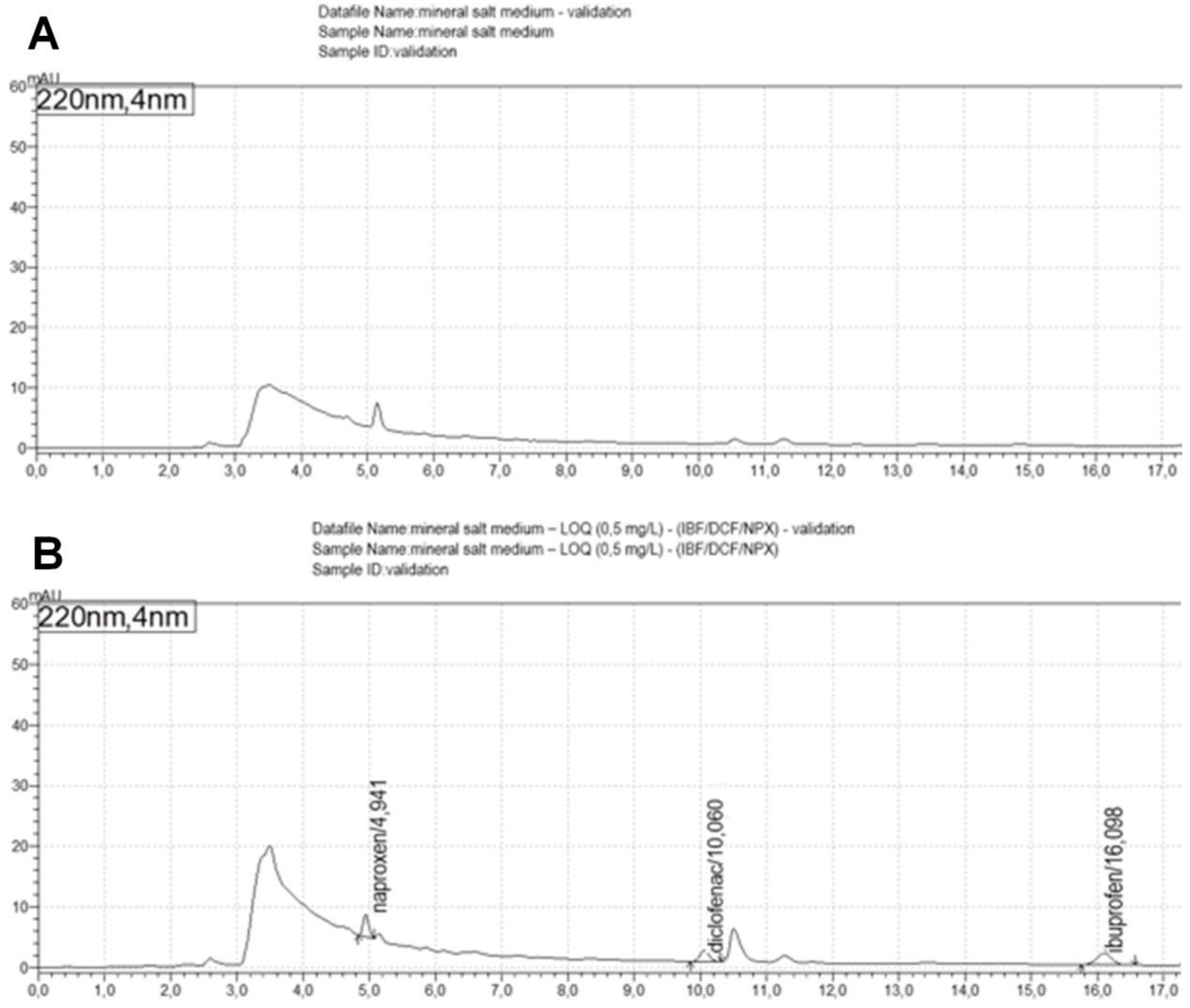

Supplementary Material Figure S6: Example chromatograms for MSM samples – control (A) and with the addition of ibuprofen, diclofenac and naproxen standards at LOQ (0.5 mg/L) levels (B) obtained during the validation procedure of the analytical method used to determine NSAID concentrations in MSM.

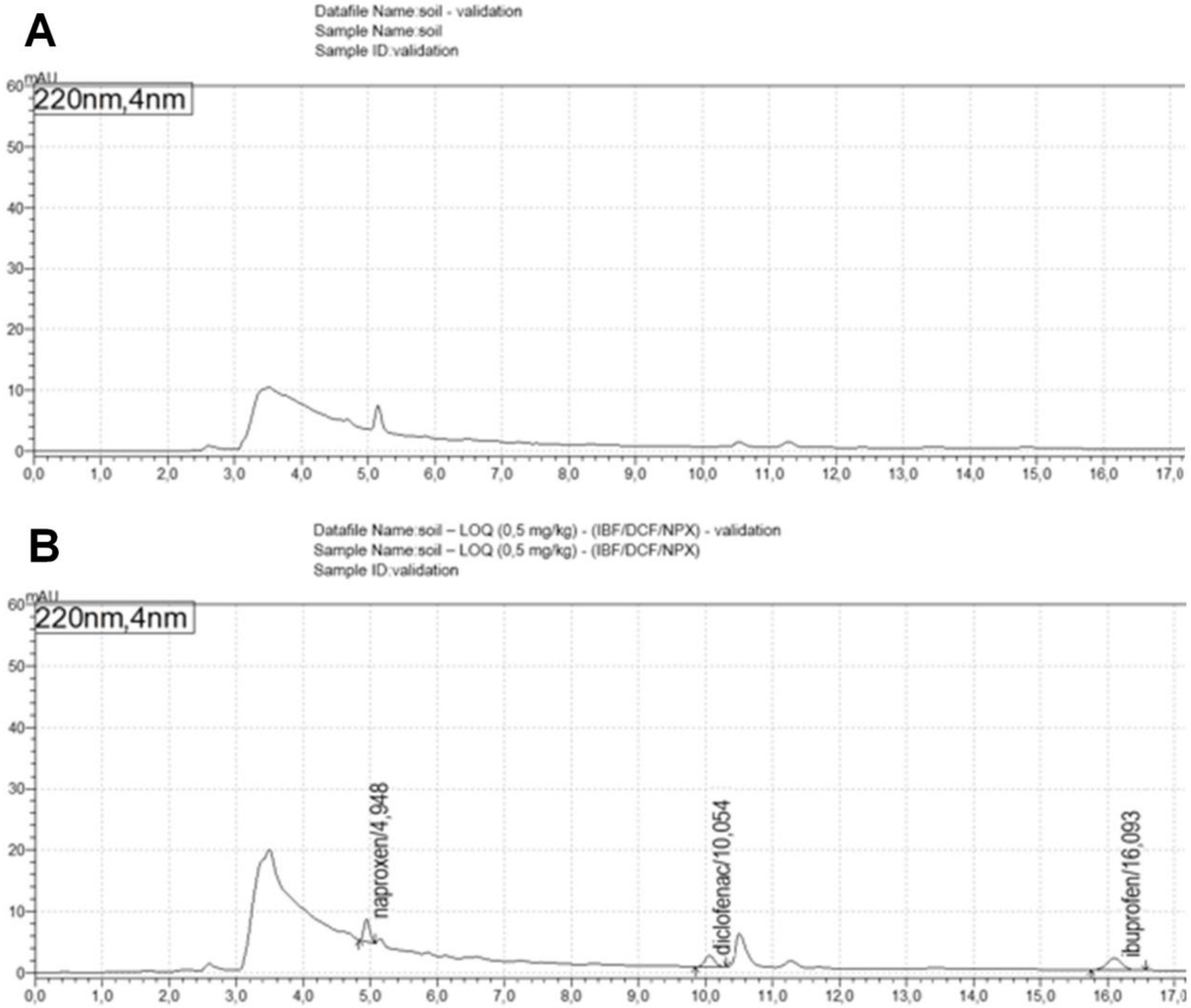

Supplementary Material Figure S7: Example chromatograms for soil samples – control (A) and with the addition of ibuprofen, diclofenac and naproxen standards at LOQ (0.5 mg/kg) levels (B) obtained during the validation procedure of the analytical method used to determine NSAID concentrations in soil.
